# Gas-Phase Collisions with Trimethylamine-*N*-Oxide Enable Activation-Controlled Protein Ion Charge Reduction

**DOI:** 10.1007/s13361-019-02177-8

**Published:** 2019-07-08

**Authors:** Margit Kaldmäe, Nicklas Österlund, Danai Lianoudaki, Cagla Sahin, Peter Bergman, Tomas Nyman, Nina Kronqvist, Leopold L. Ilag, Timothy M. Allison, Erik G. Marklund, Michael Landreh

**Affiliations:** 1 Science for Life Laboratory, Department of Microbiology, Tumor and Cell Biology, Karolinska Institutet, 171 65 Stockholm, Sweden; 2 Department of Biochemistry and Biophysics, Stockholm University, 106 91 Stockholm, Sweden; 3 Department of Environmental Science and Analytical Chemistry, Stockholm University, 106 91 Stockholm, Sweden; 4 Division of Clinical Microbiology, Department of Laboratory Medicine, Karolinska Institutet, 141 86 Huddinge, Sweden; 5 Protein Science Facility, Department of Medical Biochemistry and Biophysics, Karolinska Institutet, 171 65 Stockholm, Sweden; 6 Division for Neurogeriatrics, Department of Neurobiology, Care Sciences and Society (NVS), Karolinska Institutet, 141 83 Huddinge, Sweden; 7 Biomolecular Interaction Centre and School of Physical and Chemical Sciences, University of Canterbury, Christchurch, 8140 New Zealand; 8 Department of Chemistry – BMC, Uppsala University, Box 576, 751 23 Uppsala, Sweden

**Keywords:** Charge reduction, Protein structure, Native mass spectrometry, Gas-phase basicity

## Abstract

Modulating protein ion charge is a useful tool for the study of protein folding and interactions by electrospray ionization mass spectrometry. Here, we investigate activation-dependent charge reduction of protein ions with the chemical chaperone trimethylamine-*N*-oxide (TMAO). Based on experiments carried out on proteins ranging from 4.5 to 35 kDa, we find that when combined with collisional activation, TMAO removes approximately 60% of the charges acquired under native conditions. Ion mobility measurements furthermore show that TMAO-mediated charge reduction produces the same end charge state and arrival time distributions for native-like and denatured protein ions. Our results suggest that gas-phase collisions between the protein ions and TMAO result in proton transfer, in line with previous findings for dimethyl- and trimethylamine. By adjusting the energy of the collisions experienced by the ions, it is possible to control the degree of charge reduction, making TMAO a highly dynamic charge reducer that opens new avenues for manipulating protein charge states in ESI-MS and for investigating the relationship between protein charge and conformation.

**Electronic supplementary material:**

The online version of this article (10.1007/s13361-019-02177-8) contains supplementary material, which is available to authorized users.

**Figure:**
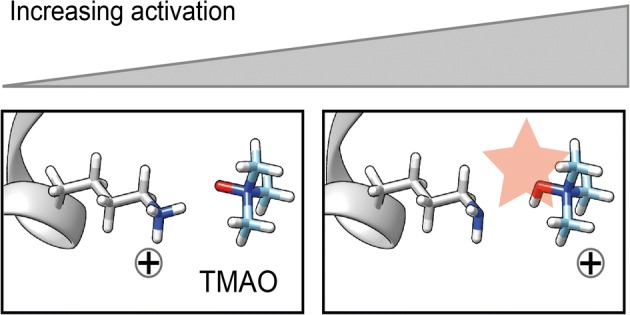
ᅟ

## Introduction

The analysis of intact proteins by electrospray ionization (ESI)-mass spectrometry (MS) relies on the generation of multiply charged ions from solution, where protein solution structure and ion charge are intricately related. The prevailing hypothesis is that the number of charges on a protein ion is largely determined by the protein’s conformation in solution [1–3]. Here, globular proteins give rise to narrow charge state distributions, whereas denatured proteins acquire a higher number of charges and a broad charge state distribution. The gas-phase conformations of the ionized proteins are thus closely correlated with charge: ions with a relatively low number of charges generally exhibit compact conformations, while highly charged ions preferentially populate extended conformations.

The number of charges on a protein ion can be manipulated through the use of supercharging or charge-reducing agents. The technique of charge reduction is often used to preserve protein conformations and interactions for MS analysis, and obtain better resolution of ions with similar masses [4, 5]. Charge reduction in ESI is most commonly carried out by adding compounds with high gas-phase basicities (GB) directly to the ESI solution [4–6], where the degree of charge reduction is believed to largely correlate with the GB of the additive and the protein [5, 7]. Alternatively, charge reduction can be achieved through gas-phase proton transfer reactions. It has been proposed that gas-phase collisions between protein ions and ammonia, methyl-, or ethylamines effectively reduce ion charges, and found that intact disulfide bonds, but not initial ion conformation, affects the proton transfer reactivity of desolvated proteins [8–10].

We have recently demonstrated that addition of the chemical chaperone trimethylamine-*N*-oxide (TMAO) in combination with collisional activation in the ion source of the mass spectrometer substantially reduces the charges of protein ions [11]. Here, we find that activation-dependent charge reduction by TMAO produces the same end charge states and arrival time distributions for denatured and native-like protein ions. Taken together, our findings indicate that charge reduction with TMAO is determined by the number and energy of the collisions experienced by the protein ions, and thus likely scales with the protein ion’s collision cross-sections (CCSs). The ability to tune the energy of the collisions and thus the degree of charge reduction by TMAO offers new opportunities to study the relationship between ion charge and gas-phase conformation.

## Results

### TMAO-Mediated Charge Reduction Is pH-Independent

**Fig. 1:**
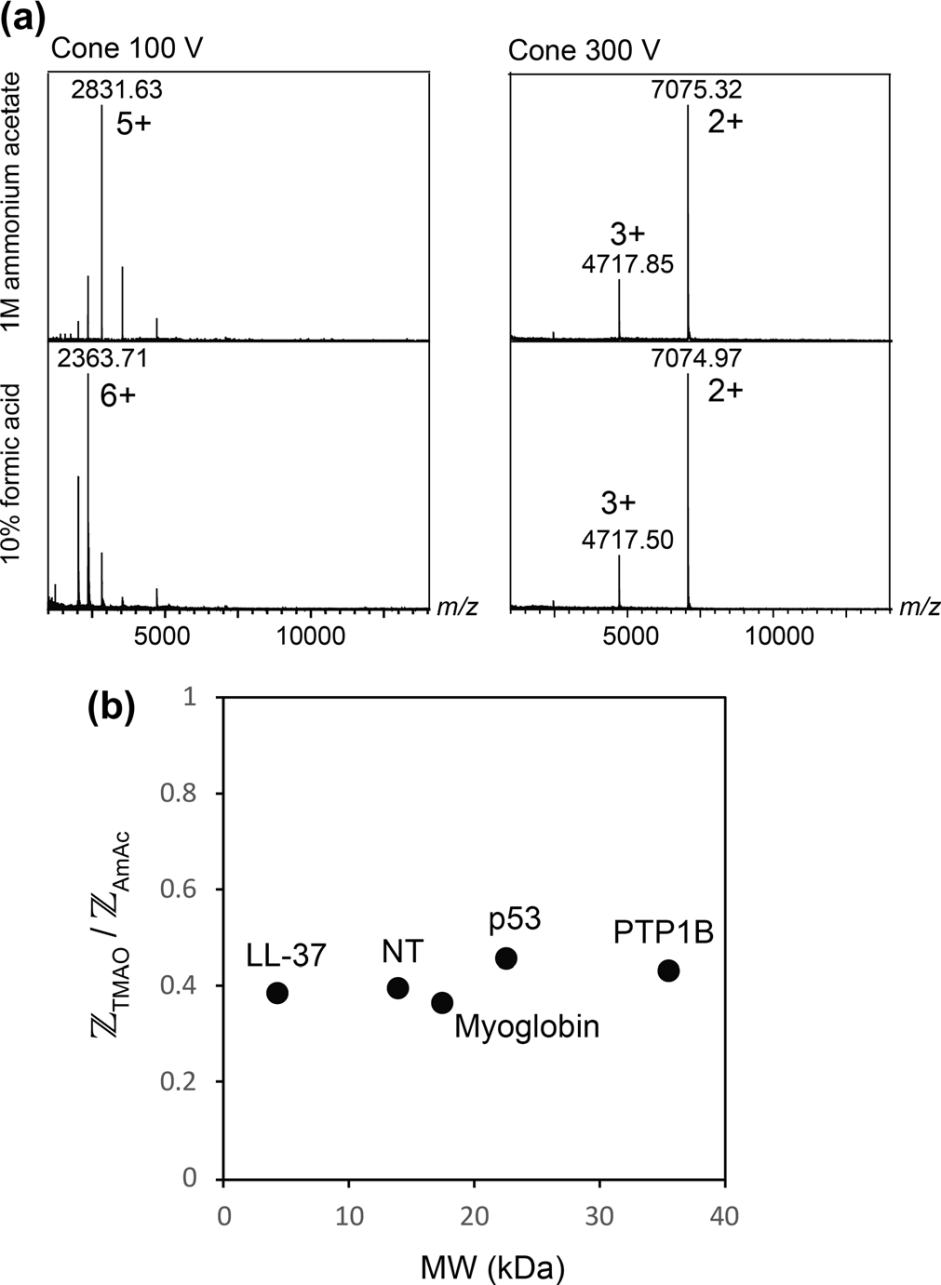
(**a**) TMAO charge-reduces above and below its pKa. Left: Mass spectra of NT in the presence of 100 mM TMAO in 1 M ammonium acetate, pH 7.5 (top) and in 10% formic acid (bottom) recorded at a cone voltage of 100 V, with the *m/z* value and charge state indicated for the most intense peak. Right: Increasing the cone voltage to 300 V to provide enhanced collisional activation reduces the charge of NT to 2.3+ and 2.2+ in ammonium acetate and formic acid, respectively. (**b**) TMAO reduces the charge of proteins by approximately 60%, as shown by the ratios between the average charge in TMAO with maximum collisional activation (Z_TMAO_), and the average charge in ammonium acetate, pH 7.5, without activation (Z_AmAc_). p53, p53 DNA-binding domain; PTP1B, phosphotyrosine phosphatase 1B

The most effective charge reduction to date has been achieved using the highly basic “proton sponges” 1,5-diazalbicyclol[4.3.0]non-5-nene (DBN), 1,8-diazabicyclol[5.4.0]undec-7-ene (DBU), and 1,5,7-triazabicyclo[4.4.0]dec-5-ene (HPP). All have a pKa of > 12, and reduce ion charges in native MS by 50–60% [5]. To characterize the charge reduction abilities of TMAO, we performed MS analysis of the highly soluble N-terminal domain (NT) from spider dragline silk (14 kDa) in 1 M ammonium acetate, pH 7.5, containing 100 mM TMAO. In the absence of collisional activation, NT had an average charge of 7.1+, similar to its average charge in the absence of TMAO (Figure S1) [11]. When we then applied collisional activation in the ion source region of the mass spectrometer by raising the cone voltage from 100 to 300 V, the average charge dropped to 2.3+, corresponding to a 61% reduction (Fig. 1a). However, unlike DBN and DBU, which achieve similar levels of charge reduction, TMAO is a weak base in solution with a pKa of 4.7. To test whether charge reduction by TMAO is dependent on the solution pH, we repeated the experiment in the presence of 10% formic acid, which results in a solution pH of about 1.5. Under these conditions in the presence of TMAO, the average charge of NT with low collisional activation dropped to 6.2+. With additional collisional activation, the average charge again dropped to 2.2+ (Fig. 1a), suggesting that the charge-reducing effect is independent of the protonation state of TMAO in solution. To test whether the degree of charge reduction is related to the molecular weight of the ions, we repeated the experiment with four proteins ranging from 4.5 to 35 kDa. The degree of charge reduction remained remarkably stable, with approximately 40% of the charge of the original ion remaining under the selected conditions (Fig. 1b).

### TMAO Produces Similar Charge States and Arrival Time Distributions for Native-Like and Denatured Proteins

**Fig. 2:**
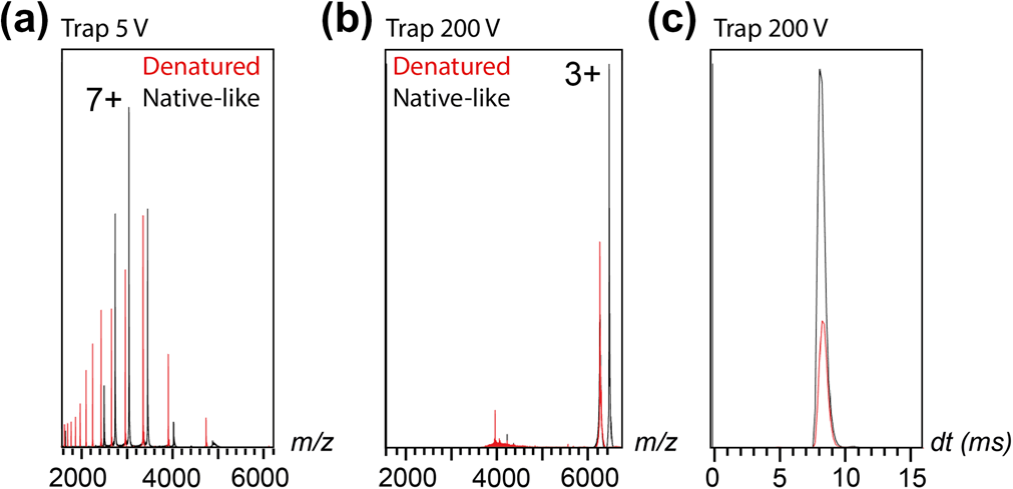
TMAO-mediated charge reduction produces the same end charge state and arrival time distribution for native-like and denatured myoglobin. (**a**) Mass spectra of myoglobin in the presence of 100 mM TMAO in 1 M ammonium acetate, pH 7.5 (black trace) or 10% formic acid (red trace) produce apo- and holo-myoglobin. (**b**) Increasing the ion trap voltage to 200 V results in an average charge of 3+ for both apo- and holo-myoglobin. (**c**) The 3+ ions of apo-myoglobin (red) and holo-myoglobin (black) shown in (**b**) have similar arrival time distributions, and hence similar CCSs

Since native and denatured proteins generate compact or unfolded protein ions, we investigated if the solution structure of the protein affects the extent of charge reduction. Myoglobin contains a non-covalently attached heme group which dissociates during denaturation and thus allows us to differentiate between folded and unfolded states by MS [12]. When subjected to ESI-ion mobility MS in 1 M ammonium acetate, pH 7.5, myoglobin retains its heme group and a narrow charge state distribution with an average charge of 7.3+ (Fig. 2). Charge reduction with 100 mM TMAO and collisional activation in the ion trap resulted in the generation of a 3+ ion as the sole charge state. Notably, even at 200 V, the highest activation level accessible by the ion trap of the instrument, we observe that > 95% of the myoglobin ions retained the heme group, indicating that the protein is able to retain parts of its native structure throughout the charge reduction process. Addition of 10% formic acid resulted in loss of the heme group and increased the average charge state of the protein to 10.8+ (Fig. 2). Strikingly, collisional activation in the presence of TMAO again resulted in a single 3+ charge state for the apo-protein. Since the lowest accessible charge state is thus independent of the protein conformation in solution, we investigated the effect of charge reduction on the gas-phase conformation. Comparison of the 3+ ions of apo- and holo-myoglobin shows near-identical arrival time distributions (Fig. 2b). Although an accurate CCS determination is not feasible for extremely charge-reduced ions [13], our data suggest that native-like and denatured protein ions exhibit highly similar CCSs upon charge reduction.

### Removing Residues with High Gas-Phase Basicity Increases TMAO-Mediated Charge Reduction

**Fig. 3:**
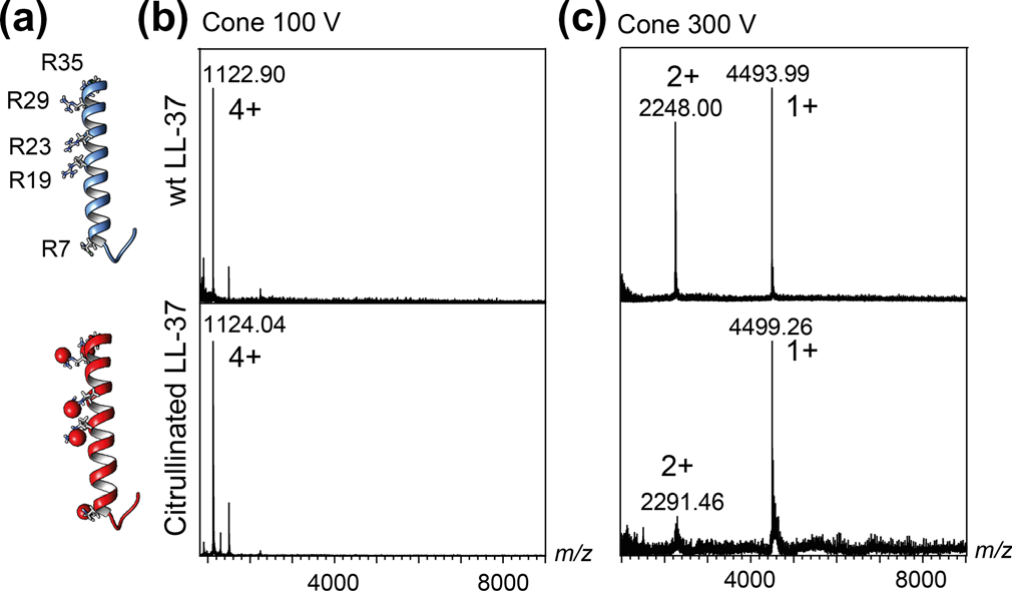
Basic residues reduce TMAO-mediated charge reduction. (**a**) Structures of wild-type and citrullinated LL-37. (**b**) Mass spectra of unmodified LL-37 (top) and with its five arginine residues neutralized by citrullination (bottom) show identical charge state distributions in the absence of collisional activation. (**c**) At a cone voltage of 300 V, citrullinated LL-37 exhibits greater charge reduction and reduced ion intensity than the unmodified peptide

It is commonly assumed that positive ESI charges predominantly, but not exclusively, reside on basic sites on a protein such as arginine and lysine side chains [2, 14]. We thus investigated whether the presence of such basic sites affects the charge reduction process. We selected the human cathelicidin peptide LL-37, which has a net charge in solution of 6+ at pH 7.5 and contains five arginine and six lysine residues in its 37-residue sequence. In 1 M ammonium acetate, pH 7.5, with 100 mM TMAO, the peptide had an average charge in the gas phase of 4+, which could be reduced to 1.5+ through collisional activation (Fig. 3). We then compared an LL-37 variant in which all five arginines were capped by citrullination, which lowers the net charge of the peptide to 1+ in solution without affecting its helical fold [15]. Like the unmodified peptide, the citrullinated form had the same charge state distribution at pH 7.5 in the presence of TMAO and low collisional activation. Increased collisional activation reduced the charge to 1+, with an overall lower signal intensity, suggesting that some of the ions may be lost as uncharged species (Fig. 3). Therefore, reducing the number of basic sites increases charge reduction by TMAO. Although basic residues play a larger role in peptide than protein charging [7], our observations indicate that the number of basic sites affects the extent of charge reduction by TMAO.

## Discussion

In the present study, we show that TMAO in combination with collisional activation produces the same end charge state for folded and denatured proteins independent of solution pH. Strikingly, we find that the weak base TMAO (GB 953 kJ/mol) in combination with collisional activation is as effective for charge reduction as highly basic proton sponges with significantly higher GB values (1006–1022 kJ/mol) [5]. In the absence of collisional activation, on the other hand, insignificant charge reduction by TMAO is observed, despite its GB being higher than that of common charge-reducing agents like dimethyl sulfoxide (853 kJ/mol) or imidazole (909 kJ/mol). Although we and others previously showed that TMAO, trimethylamine, and triethylamine adducts can dissociate as charged species [11, 16], our observations cannot be explained based on proton affinities alone.

In a series of studies, Smith and co-workers demonstrated that gas-phase collisions of desolvated proteins with ammonia, methylamines, and ethylamines resulted in effective charge reduction that roughly correlated with the number of arginines available for protonation [8–10, 17]. They were able to show that protein ions generated from denaturing and native-like ESI solutions [10], but not under reducing and non-reducing conditions [8, 10], yielded the same end charge states. These findings are in good agreement with the observations made for TMAO. Here, the high TMAO concentration present in the ESI solution likely facilitates collisions with protein ions in the source and trap regions of the mass spectrometer. The fact that we find a strict dependence on collisional activation suggests that we are able to tune the energy and intervals of these collisions to determine the degree of charge reduction at a given TMAO concentration. Similar, but not as pronounced, effects were previously reported for trimethylamine and triethylamine [16].

In the original studies by Smith and co-workers, it was concluded that denatured and native-like protein ions display very similar proton transfer reactivity thus likely have similar gas-phase conformations [10]. This led us to analyze the conformations of charge-reduced myoglobin by ion mobility. We found that the 3+ ions of both denatured apo- and native-like holo-myoglobin have identical drift times. In line with results from post-ionization charge reduction studies [18], this suggests that with progressive charge reduction, denatured and native-like ions adopt gas-phase conformations with similar CCS. As their CCSs converge, the ions likely experience a similar number of collisions, and thus reach a similar final charge state regardless of their starting conformation. It is then tempting to speculate that intact disulfide bonds restrict the number of gas-phase conformers, and thus, ions produced under reducing and non-reducing conditions may have different CCSs and thus reach different end charge states.

In summary, activation-dependent charge reduction of protein ions by TMAO as demonstrated here can provide a simple means to manipulate the charge state and gas-phase conformations of protein ions independent of solution conditions, opening new avenues for the study of their structures in the gas-phase.

## Electronic Supplementary Material

ESM 1 (PDF 101 KB)

## Abbreviations

*GB*: Gas-phase basicity
*TMAO*: trimethylamine-*N*-oxide
