# Supplementary material for: Gas-Phase Collisions with Trimethylamine-N-Oxide Enable Activation-Controlled Protein Ion Charge Reduction
Source: J Am Soc Mass Spectrom. 2019 Jul 8;30(8):1385–8. doi: 10.1007/s13361-019-02177-8 (PMC6669196; doi:10.1007/s13361-019-02177-8)
Supplement: Supplementary file 1 — (PDF 101 KB) [file 13361_2019_2177_MOESM1_ESM.pdf]

Supplementary information to

Gas-phase collisions with trimethylamine-*N*-oxide enable activation-controlled protein ion charge reduction

Margit Kaldmäe, Nicklas Österlund, Danai Lianoudaki, Cagla Sahin, Peter Bergman, Tomas Nyman, Nina Kronqvist, Leopold L. Ilag, Timothy M. Allison, Erik G. Marklund, Michael Landreh

Materials and Methods

Protein preparation

All chemicals and sperm whale myoglobin were purchased from Sigma. Unmodified and citrullinated LL-37 were purchased from Innovagen (Lund, Sweden). His_6-NT from major ampullate spidroin 1 from *Euprosthenops australis* with the D40K/K65D mutation for increased stability was expressed and purified as described (1) and stored at -20 °C in 20 mM phosphate buffer, pH 7.5, until analysis. C-terminally truncated human PTP1B (residues 1-301) was purified as described (2) but without the size-exlcusion step.
For the DNA-binding domain of human p53, a DNA fragment covering residues 94-292 was cloned into the expression vector pNIC28-Bsa4, adding an N-terminal 6xHis-tag followed by a TEV protease recognition site. BL21(DE3) T1R pRARE2 cells (Sigma) were transformed with the p53(94-292) pNIC28-Bsa4 construct and 1.5 l TB (supplemented with 8 g/L Glycerol, 50 µg/mL Kanamycin, 34 µg/ml Chloramphenicol) cultures were started from overnight cultures (grown in the same medium at 30 °C). The cultures were grown at 37 °C until expression was induced with IPTG (0.5 mM) at an OD_{600} of 2 and allowed to continue for 16 hrs at 18 °C. Cells were harvested by centrifugation and re-suspended in lysis buffer (50 mM Tris, 800 mM NaCl, 10% glycerol, 10 mM imidazole, 0.5 mM TCEP, pH 8.0, supplemented with 5 µl Benzonase Nuclease (Sigma), Complete EDTA free protease inhibitor cocktail tablet (Roche) and then frozen at -80 °C. The thawed re-suspended cells were lysed by sonication and the cell debris removed by centrifugation and filtration of the supernatant through 0.45 µm filters. Chromatography was carried out using an Äkta Xpress (GE Healthcare) at 8 °C. A HisTrap (GE Healthcare) column was loaded with the supernatant and then washed 50 mM TRIS, 800 mM NaCl, 10% glycerol, 0.5 mM TCEP, pH 8.0 containing 10 mM and then 50 mM imidazole before being eluted with Elution Buffer (50 mM TRIS, 800 mM NaCl, 10% glycerol, 500 mM imidazole, 0.5 mM TCEP, pH 8.0). The eluate was loaded on to a HiLoad 16/60 Superdex 75 (GE Healthcare) column in Gel Filtration buffer (50 mM TRIS, 800 mM NaCl, 10% glycerol (V/V), 2 mM TCEP, pH 8.0). The His-tag was removed by the addition of His-tagged TEVsh protease in a ratio of 1:30 (protease:target protein) at 4 °C over-night and the protein isolated by passing through a 5 ml HisTrap column. The p53(94-292) protein was concentrated to 7 ml of 1.9 mg/mL using a Vivaspin concentrator (Sartorius) and stored at -80 °C.

Mass spectrometry

Proteins were diluted to a final concentration of approximately 20 µM in 1 M ammonium acetate, pH 7.5, and desalted using Biospin 6 microcentrifuge columns (BioRad). TMAO was added from a 1 M stock in dH_2O and subjected to MS analysis immediately.
Mass spectra were acquired on a Micromass LCT ToF modified for analysis of intact protein complexes (MS Vision, The Netherlands) equipped with an offline nanospray source. ESI capillaries were purchased from Thermo. The capillary voltage was 1.5 kV and the RF lens 1.5 kV. Collisional activation was controlled by setting the cone voltage to 100

or 300 V. The pressure in the ion source was maintained at 9.0 mbar. Spectra were visualized using MassLynx 4.1 (Waters).
Ion mobility MS spectra were acquired on a Synapt G2S TWIMS equipped with an offline nanospray source. The capillary voltage was 2 kV and the source temperature was maintained at 120 ºC and the sample cone at 80 V. Collisional activation was performed by setting the trap voltage to 5 V or 200 V. The transfer voltage was 2 V. Trap and drift gas was Nitrogen with a flow of 10 mL/min in the ion trap and 50 mL/min in the IMS cell. The pressures were: backing 3.27 mbar, source 5.76 e-3 mbar, trap 3.60 e-2 mbar, IMS 1.24 mbar. The IMS wave height was 10 V and the wave velocity was 600 m/s. The trap DC bias was 45 V and the IMS DC bias was 3 V. Data were visualized using MassLynx 4.1 (Waters).

Data analysis
Average charge states were determined by smoothing the spectra 10× in MassLynx and calculating intensity-weighted averages using Microsoft Excel. Gas phase basicities were taken from the NIST Chemistry WebBook (http://webbook.nist.gov/chemistry). Figures were prepared using Adobe Illustrator 6.

References
(1) Kronqvist N, Sarr M, Lindqvist A, Nordling K, Otikovs M, Venturi L, Pioselli B, Purhonen P, Landreh M, Biverstål H, Toleikis Z, Sjöberg L, Robinson CV, Pelizzi N, Jörnvall H, Hebert H, Jaudzems K, Curstedt T, Rising A, and Johansson J.(2017) Efficient protein production inspired by how spiders make silk. Nat. Commun. 8:15504. doi: 10.1038/ncomms15504
(2) Krishnan, N, Koveal, D, Miller, DH, Xue, B, Akshinthala, SD, Kragelj, J, Jensen, MR, Gauss, CM, Page, R, Blackledge, M, Muthuswamy, SK, Peti, W, and Tonks, NK. (2014) Targeting the disordered C terminus of PTP1B with an allosteric inhibitor. Nat Chem Biol. 10:558-66.

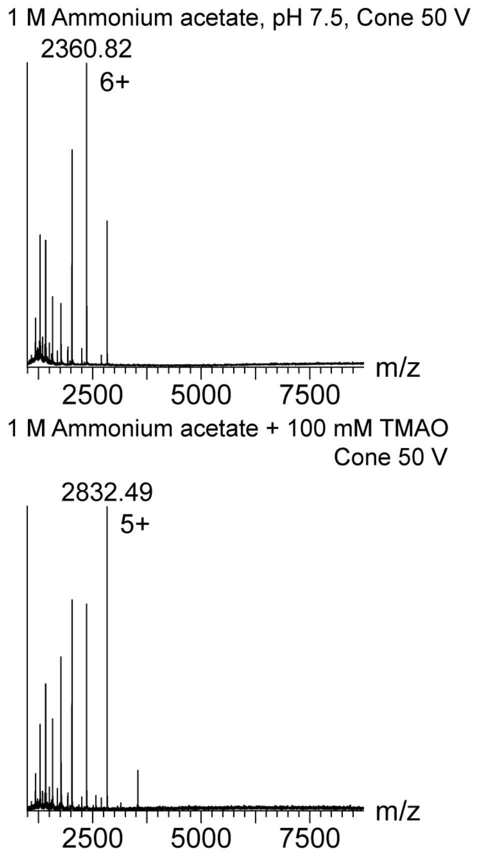

Figure S1. MS spectra at minimum collisional activation (cone voltage of 50 V) show similar charge state distributions for NT in the absence (top) or the presence (bottom) of 100 mM TMAO.
